# Antibacterial Activity of Eucalyptus Essential Oils in Both Culture Media and Coated Materials

**DOI:** 10.1155/ijm/2756030

**Published:** 2025-05-26

**Authors:** Théoneste Muhizi, Gervais Manizabayo, Colores Uwamariya, Jean Bosco Nkuranga, Daniel Umereweneza

**Affiliations:** Department of Chemistry, School of Sciences-College of Science and Technology-University of Rwanda, Kigali, Rwanda

**Keywords:** bioactivity, chemical composition, essential oils, eucalyptus species, hydroxypropyl cellulose films

## Abstract

Different factors, including microbial resistance, have led to food contamination and increased human intoxication risks. Therefore, finding new adequate methods to fight against bacterial development is of interest. This study investigates the efficiency of eucalyptus essential oils to inhibit the growth of *Escherichia coli*, *Klebsiella pneumoniae*, *Listeria innocua*, *Salmonella typhimurium*, *Staphylococcus aureus,* and *Streptococcus pyogenes.* Oils were extracted from eucalyptus leaves by hydrodistillation and chemically analyzed on a coupled gas chromatography–mass spectrometer. The antibacterial activity was assessed in both culture media and film models. Results indicated that eucalyptus oils were rich in different chemicals including *α*-pinene in oils from *E. obliqua* (89.3%), *E. grandis* (39.9%), and *E. microcoris* (29.6%); p-cymene in *E. camaldulensis* (40.1%) and *E. grandis* (25.8%); and eucalyptol in *E. viminalis* (88.3%), *E. crebra* (84.6%), *E. polyanthemus* (80.7%), *E. melliodora* (78%), *E. maiden* (74.8%), and *E. globulus* (70.4%) oils. Terpinen-4-ol was found in *E. anceps* (5.7%) and *E. camaldulensis* (5.6%) while oils from *E. anceps* contained *β*-phellandrene (4.7%), cis- and trans-piperitols (6.5%), (cis)-p-(2 menthen)-1-ol (8.3%), geraniol (19.9%), and piperitone (10.5%). In comparison with a known antibiotic, azithromycin, essential oils from *E. grandis* and *E. anceps* exhibited significant antimicrobial efficacy against the growth of all tested microorganisms. The antibacterial efficiency from these oils was found to be higher than other essential oils tested (*p* < 0.05), with minimum inhibitory concentration and minimum bactericidal concentration ranging from 0.5 to 7 and 2.5 to 20 *μ*L/mL, respectively. Yet, at all concentrations tested (5–15 *μ*L/mL), hydroxy-propyl cellulose (HPC) films containing these oils indicated significant inhibition efficacy of the growth of bacteria compared to HPC films (*p* < 0.001). Further study on *E. grandis* and *E. anceps* oils is needed for their various valorization.

## 1. Introduction

The use of biocides to fight against harmful microorganisms has been for a long time [1, 2]. These products are used in different domains including medicines, agriculture, food, and materials preservation for controlling harmful microorganisms and thus maintaining human life [2]. *Salmonella, Listeria, Staphylococcus, Bacillus cereus, Escherichia coli, Clostridium botulinum*, and *Candida albicans* are among several harmful microbes causing different human illnesses and death when absorbed [3, 4]. For example, in 2015, cases of diseases and death caused by *Salmonella typhi* were estimated to be between 11–21 million and 148,000–161,000, respectively, while in 2019, 9.2 million typhoid fever cases and 110,000 deaths occurred worldwide [5–7]. Other reports indicated the existence of different microbial diseases in both developing and developed countries [8, 9], thus posing a big concern to humanity. Yet, the danger associated with harmful microbes was frequently linked to their resistance to existing antimicrobial drugs and their toxicity towards nontargeted organisms [2, 6, 10, 11–13]. Studies conducted in the food domain indicated that some of these products used as food additives and preservation exhibited toxicity [14–16]. Consequently, new efficient biocides are needed to deal with the situation. Many natural products, including essential oils from eucalyptus species, are of interest because of their known use in traditional medicines and biodegradation characteristics [17–19]. These oils from eucalyptus species indicated promising biological activity and may constitute alternative starting materials for new biocides' discovery. Represented by more than 600 species distributed elsewhere in the world as the most planted pulpwood species, the *Eucalyptus* genus is classified in the family of Myrtaceae and is native to Australia [20, 21]. The genus was planted in Rwanda in the early 1900s because of its adaptation to different regions. It is now considered the most abundant plantation in Rwanda. In this country, in 2015, eucalyptus species occupied 78% of all plantations [22, 23]. The accumulation in the soil of the chemical products from its leaves makes them an environmental hazard, since they enter the soil, increase its acidity, and lead to allelopathic effects [24, 25]. However, if well exploited, these products can be useful to humanity. One of these products, essential oils, indicated different biological activities including insecticidal, herbicidal, fungicidal, acaricidal, bactericidal, and nematicidal activities [26–28]. Furthermore, local traditional healers revealed that eucalyptus leaves mixed with either one or several leaves from *Guizotia scabra*, *Lagenaria abyssinica*, *Acacia abyssinica*, *Allium sativum*, *Cupressus* spp., *Tropaeolum majus*, *Aloe* spp., and *Helichrysum nforskahlii* are efficient in treating flu, intestinal worms, cough, malaria, and diarrhea. Moreover, previous studies on *E. obliqua* indicated its efficacy in treating malaria, fever, inflammatory diseases, diabetes mellitus, and liver disorders [29–32]. Further, previous studies indicated that these oils may be incorporated in film coating materials, and depending on their chemical components, they may increase their biological activity [33, 34]. Despite these reported interesting biological activities that can be a good alternative to valorize these leaves, essential oils from all eucalyptus species are far from being extracted, characterized, and adequately used. For example, Ruhande Arboretum, which is located in the Southern Province of Rwanda, possesses 69 eucalyptus species [35], and only 22 eucalyptus species have been studied for their essential oils' composition [36]. Knowing that the concentration of natural products from plants is seriously affected by different factors including soil, weather, time, and others [37–40] and that few species of that tree in Rwanda were studied [36], this study was aimed at exploring the efficacy of the remaining eucalyptus species for further applications.

## 2. Materials and Methods

### 2.1. Chemicals

Distilled water culture media includes the following: tryptose broth (Difco 262200), nutrient broth (Difco 234000), hydroxy-propyl cellulose (HPC) 99% (Merck), ethanol 95.5% (FisherScientific), Tween 80 (LobaChemie Pvt. Ltd), tetradecane 99% (Fluca AG), n-hexane (99%, Rankem), anhydrous sulfate (LobaChemie Pvt. Ltd), and dimethyl sulfoxide (99.9%, Wagtech International Ltd). These also include *α*-pinene (Fluka chemicals), myrcene (Gambetta, France), eucalyptol (Prolabo), *α*-phellandrene (Eastman organic chemicals), p-cymene (Fluka chemicals), *α*-terpineol (Gambetta, France), limonene (Sigma chemical), and azithromycin (Alice Pharma PVT LTD, India).

### 2.2. Plant Materials

Leaves of eucalyptus species, *E. anceps*, *E. camaldulensis*, *E. grandis*, *E. globulus*, *E. melliodora*, *E. microcoris*, *E. obliqua*, *E. crebra*, *E. polyanthemus*, *E. viminalis*, and *E. maideni* were collected from the mature trees with more than 10 years old, in the dry season of August 2022 from Ruhande arboretum forest (29°44⁣′ E and 2°36⁣′ S) located in Huye District, Southern Province of Rwanda. Species were identified by their geographic location in defined and labeled plots and were confirmed by a botanist of the Biology Department, University of Rwanda. Their voucher specimens with numbers URNHR, S00018, S00008, S00131, S00006, S00032, S00060, S00142, S00024, S00181, S00130, and S00086, respectively, were deposited in the Herbarium of the CoEB, Rwanda.

### 2.3. Bacterial Strains and Culture Media

Bacteria, *Staphylococcus aureus* (URML22-0018), *Streptococcus pyogenes* (URML22-0012), *Listeria innocua* (URML22-0001), *Klebsiella pneumoniae* (URML22-0011), *Escherichia coli* (URML22-0184), and *Salmonella typhimurium* (URML22-0022) used in this study were isolated and characterized by the Microbiology Laboratory of the University Hospital, University of Rwanda. Tryptose broth was used to revive *S. aureus*, *S. pyogenes*, and *L. innocua*, while nutrient broth was used for *E. coli*, *K. pneumoniae*, and *S*. *typhimurium*.

### 2.4. Extraction of Essential Oils

Leaves were washed with distilled water and left to dry at room temperature (25°C) for 3 h. Then, 600 g of each were subjected to hydrodistillation using a Clevenger-type apparatus for 6 h, and this process was repeated three times for each species. The obtained essential oils mixed with some water quantity were put in a separating funnel, let to stand for 20 min, and then separated with water. Thereafter, they were dried with anhydrous sodium sulfate and weighed before being kept in opaque vials at 0°C for further studies. The yield of essential oil was calculated in percentage through the mathematical formula:

 $$\begin{array}{c} \text{Yield}=\frac{\text{Mass of the dried essential oil}}{\text{Mass of the Leaves}}\times 100. \end{array}$$

### 2.5. Characterization of Essential Oils

The obtained essential oils were analyzed on a Thermos Finnigan GC fitted with a BP 21 SGE FFAP column (25 m × 0.22 mm) with a flame ionization detector (FID). The temperature in the oven was maintained at 50°C for 1 min, then increased progressively to 200°C at a rate of 4.5°C·min^−1^, and kept constant at 200°C for 5 min before sample injection. Helium was used as carrier gas at a 1.2 mL/min flow rate. To increase the sensitivity of minor components, 10% of each essential oil was dissolved in hexane before injection into the gas chromatography (GC) spectrometer. A volume of 1 *μ*L of the prepared solution was injected at 200°C, and the percentage composition of components was obtained by normalizing GC peak areas with 0.015 mg·mL^−1^ of tetradecane (free of olefin, ≥ 99% purity, Fluka AG) used as reference. The injection of essential oil was done in triplicate, and the mean relative percentage that was obtained was recorded. With the same experimental conditions used on GC, regarding column, temperature in the oven, and carrier, the analysis was continued on coupled gas chromatography–mass spectroscopy, GC/MS Ultra DSQ Thermo equipment, to complete and confirm GC data. With this analysis, the components from the GC column were fractionated in a mass spectrometer with m/z 5-500 mass range. Scanning was done at an interval of 0.5 sec with a scanning speed of 1000 amu/s and an ionization voltage of 70 eV. Quantification of different constituents, expressed in percentage, was done by peak area normalization measurements. The identification of essential oils' components was possible through comparison of their retention time (RT), retention index (KI), and mass spectra (MS) with those obtained from authentic samples, computer library mass spectra, and literature. The quantification of different constituents, expressed in percentage, was done by peak area normalization measurements.

### 2.6. Antimicrobial Assessment

#### 2.6.1. Inoculum Preparation

To have a bacterial inoculum, two colonies forming unity from a reviving Petri dish were suspended in 10 mL of the suitable culture broth and incubated at 37°C for 24 h. Thereafter, 1 mL of the obtained suspension was diluted with 9 mL of physiological saline and incubated again at 37°C for 18 h. The obtained strain solution was diluted 10^3^ times with physiological saline, and a 10^4^ bacterial load suspension was obtained and ready for further assessment.

#### 2.6.2. Disk Diffusion Method

The antimicrobial activity was tested using the modified disk diffusion method [41–44]. For this, 20 mL of the specific agar culture media for *L. innocua*, *S. pyogenes*, *S. aureus*, *S. typhimurium*, *K. pneumoniae*, and *E. coli* was put into each Petri dish. Thereafter, 100 *μ*L of a diluted 18 h fresh inoculum containing 10^3^ bacterial loads was carefully spread on the surface of the solid medium. After 30 min, the water was completely absorbed and the culture media dried, and 6-mm diameter sterilized cellulosic disks were gently deposited on the inoculated media. Then, a volume of 10 *μ*L of pure essential oils was gently deposited onto the disk using a micropipette. Positive control experiments were concurrently realized using 10 *μ*L of azithromycin 40 mg/mL as a known commercial drug. Thereafter, plates were incubated at 37°C for 24 h. Positive results were indicated by clear zones surrounding the disks, and their diameters were measured and recorded. Means obtained from triplicate tests were considered as final results. The same methodology was applied to test the antibacterial activity of some pure chemical compounds found in essential oils, such as *α*-pinene, myrcene, eucalyptol, *α*-phellandrene, p-cymene, *α*-terpineol, and limonene. Instead of essential oils, 10 *μ*L of a pure component was deposited on the disks to realize this test.

#### 2.6.3. Determination of the Minimum Inhibitory Concentration (MIC) and the Minimum Bactericidal Concentration (MBC)

Both broth dilution and counting methods previously described [45–47] were used to determine MIC and MBC, respectively. Essential oils with higher antibacterial activity were selected for the determination of MIC and MBC. A colony of each tested bacterium was isolated and inoculated in sterile tubes containing 10 mL of tryptose broth or nutrient broth, depending on the bacterial strain. The obtained suspension was incubated at 37°C for 24 h. Then, 1 mL of the inoculated medium was added to 9 mL of the broth and incubated for 18 h at the same temperature. Using a spectrophotometer (Spectronic 20, Milton Roy Company) set at a wavelength of 450 nm, optical activity from the bacteria suspension was fixed at 0.5, corresponding to 10^6^ bacterial load. For this, the 18-h fresh bacterial strains were diluted 10^3^, 10^5^, 10^3^, 10^4^, and 10^6^ times with physiological saline. Then, the diluted essential oils, with concentrations ranging from 0.5 to 75 *μ*L mL^−1^ in 10 mL of tryptose or nutrient broth, received 100 *μ*L of the diluted microbial strain, and the obtained suspension was incubated as described above. After shaking, a volume of 100 *μ*L from each test tube was spread on a Petri dish with a specific agar medium before incubation at 37°C for 24 h. The tube with the lowest concentration of essential oil not indicating any visible colonies forming, compared to the control test tube, was taken as the MIC. Afterward, to determine MBC, incubation of all bacterial strains in the negative test tubes was maintained for 2 days (48 h) at 37°C and bacteria were revived in tryptose or nutrient broths. Then, 100 *μ*L from each negative test tube was gently spread on specific agar culture media and incubated at 37°C for 24 h. The obtained number of colonies was compared to that in control experiments. The tube with the lowest concentration, which did not indicate any colonies of bacteria, was considered as MBC of the concerned essential oil.

### 2.7. Antibacterial Activity of Film Materials

#### 2.7.1. Preparation of HPC and EO-HPC Films and Characterization

Due to its nontoxicity, its capability to release drugs, and its water solubility [48, 49], HPC was used as the starting material to make films. HPC and two higher bioactive essential oils from *E. grandis* and *E. anceps* were chosen to prepare both HPC and essential oils (HPC–essential oil from *E. grandis*, HPC-Eg, and HPC–essential oil from *E. anceps*, HPC-Ea) films. The methods [50] were slightly changed and used in this study. Using an IKA KS 130 apparatus, a mass of 9 g of HPC was shaken together with 200 g of water and 100 g of ethanol (95.5%) for 1 h and often degassed under a vacuum pump. The obtained viscous product was poured into a Petri dish and left to dry in a sterilized laminar flow for 12 h. The obtained films were then kept at a relative humidity of 50% and a temperature of 24°C for 7 days before being evaluated for their antibacterial activity. For the preparation of essential oils–HPC films, different quantities of essential oils from *E. grandis* and *E. anceps*, previously stabilized by 0.5 mL of Tween 80 in 10 mL of a given essential oil, were added before final concentrations of 5, 10, and 15 *μ*L mL^−1^ and the procedure was continued as done on HPC films. As a negative control, films containing 0.05% of Tween 80 (HPC-T80) were also concurrently prepared and tested. Formed films were characterized by their color and thickness. The color was determined by simple observation, and the thickness by a spiral micrometer.

#### 2.7.2. Assessment of the Antibacterial Activity of Film Materials

The coating method was used to assess the antibacterial activity associated with HPC, HPC-Eg, HPC-Ea, and TW-HPC films. A volume of 100 *μ*L of an 18-h microbial suspension with a 10^2^ microbial load was deposited and gently spread on the culture medium. Thereafter, the films to be tested were deposited on the surface medium and then incubated at 37°C for 48 h. After that, the colony-forming units (CFU) were determined in each experiment and compared to those obtained from the control experiment conducted with plates containing films without essential oils. All these experiments were conducted in triplicate, and the obtained percentage of inhibition indicated the effectiveness of each tested film.

### 2.8. Statistical Analysis

One-way analysis of variance (ANOVA) was used to conduct a statistical analysis of the antimicrobial activity from different tests. If the probability (*p*) found was less than the significance threshold of 0.05 (*p* ≤ 0.05), then the difference between antibacterial activities was considered significant. A probability greater than 0.05 (*p* > 0.05) was taken as no significant difference was observed between the two compared datasets. This analysis was completed by the least significant difference (LSD) test, where the sensitivity of different bacterial strains and the antibacterial efficiency of drugs were compared. R software, Version 4.3.3, was used to conduct these analyses.

## 3. Results

### 3.1. Essential Oils' Yields

Depending on the eucalyptus species, the obtained yields ranged from 0.47% to 2.2% (*w*/*w*) (Table 1). The highest yield was from the leaves of *E. globulus* (2.2%) followed by *E. microcoris* (2.12%, *w*/*w*) while the lowest yield was from *E. obliqua* leaves (0.25%, *w*/*w*).

### 3.2. Chemical Composition of the Essential Oils

Through GC/MS analysis, 28 components were identified from 11 essential oils, and their concentrations depend on eucalyptus species (Table 2). Eucalyptol was the most frequent component in many essential oils studied: *E. globulus* (70.4%), *E. melliodora* (78.0%), *E. microcorys* (57.1%), *E. crebra* (84.6%), *E. polyanthemus* (80.7%), *E. viminalis* (88.3%), and *E. maideni* (74.8%), while p-cymene (40.1%) was found in the essential oil from the leaves of *E. camaldulensis* as the main component. Still, in this oil, eucalyptol, *α*-pinene, terpinolene, terpinen-4-ol, and cryptone were found in the respective quantities of 18.2%, 3.0%, 4.6%, 5.6%, and 6.1%. In the essential oils from the leaves of *E. grandis*, *α*-pinene, p-cymene, and eucalyptol were detected in the percentages of 39.9%, 25.8%, and 14.2%, respectively. The essential oil from *E. anceps* showed the presence of *β*-myrcene (11.1%), geraniol (19.9%), *β*-phellandrene (4.7%), terpinen-4-ol (5.7%), cis–trans-piperitols (6.5%), (cis)-p-(2-menthen)-1-ol (8.3%), and piperitone (10.5%) while that from *E. obliqua* indicated a high percentage in *α*-pinene (88.3%) and in eucalyptol with a percentage of 6.5%.

### 3.3. Antibacterial Activity of Essential Oils

The antibacterial assessment of essential oils revealed that they exhibited different antibacterial activities, and these results were expressed by various inhibition diameters (Table 3). The analysis of the results by ANOVA and LSD test indicated that for the inhibition of the *E. coli* growth, the essential oil from *E. grandis* exhibited comparable antibacterial activity with azithromycin used as a positive control (*p* > 0.5). In addition, the same essential oil and that from *E. anceps* indicated more significant antibacterial activity than other essential oils tested (*p* < 0.05). Essential oils from *E. obliqua* and *E. camaldulensis* exhibited moderate comparable antibacterial efficiency against the growth of *E. coli*, which was greater than that from *E. polynathemus*, *E. maiden*, *E. crebra*, and *E. melliodora*. Essential oils from *E. globulus*, *E. microcorys*, and *E. viminalis* exhibited less anti-*E. coli* activity compared to others. The same observation was done on *S. typhimurium*, where essential oils from *E. grandis* and *E. anceps* exhibited higher antibacterial activity against the growth of *S. typhimurium* than other essential oils, while *E. microcorys* and *E. viminalis* were the least active. For *L. innocua* and *S. aureus*, *E. grandis* and *E. anceps* were more active than *E. camaldulensis*, *E. obliqua*, and *E. maiden*. The latter were followed by *E. crebra*, *E. globulus*, *E. polyanthemus*, *E. melliodora*, and *E. microcorys*, exhibiting the same effect on the growth of these bacteria, while *E. viminalis* was the least active drug. Furthermore, essential oils from *E. grandis* and *E. anceps* indicated their efficiency in inhibiting the growth of *S. pyogenes* and *K. pneumoniae*, where their activity was close or even higher than that of azithromycin (LSD test). Once again, *E. viminalis* indicated the least antibacterial activity against these two microbes. Briefly, the LSD test indicated that for most bacteria, the antibacterial efficiency of essential oils varied as follows: *E. grandis* ≥ *E. anceps* > *E. camaldulensis, E. obliqua* > *E. maiden, E. globulus, E. crebra, E. polyanthemus* > *E. microcorys, E. melliodora, E. viminalis*. In some cases (*S. aureus*, *S. pyogenes*, and *K. pneumoniae*), essential oils from *E. grandis* and *E. anceps* exhibited higher antibacterial activity than azithromycin used as a positive control. According to the LSD test, no significant difference was found between the sensitivity of bacteria towards essential oils from *E. viminalis*, *E. microcorys*, and *E. maideni*. Besides, the analysis indicated that *S. aureus* strains were the most sensitive towards some oils, including *E. grandis*, *E. anceps*, *E. camaldulensis*, *E. obliqua*, *E. polyanthemus*, *E. crebra*, and *E. globulus*. *K. pneumoniae* and *S. pyogenes* indicated the highest sensitivity to the oils from *E. globulus* and *E. meliodora* and *E. anceps*, respectively. Meanwhile, *S. typhimurium* indicated the highest resistance to most of the essential oils tested. Antibacterial tests were conducted with selected individual components of the essential oils against the growth of *E. coli*, *S. typhimurium*, *L. innocua*, *S. aureus*, *S. pyogenes*, and *K. pneumoniae*, indicating that *α*-terpineol exhibited an antibacterial activity, which is more statistically significant (*p* > 0.1) than that of azithromycin. The same observation was done with *α*-pinene against the growth of *E. coli.* Azithromycin indicated more significant antimicrobial activity than other components, such as myrcene, eucalyptol, *α*-phellandrene, p-cymene, and limonene (*p* < 0.05) (Table 4). For *E. coli* and according to the LSD test, drugs with moderate antibacterial activity were limonene and eucalyptol. For all other bacteria tested, myrcene was the least effective, after *α*-phellandrene, eucalyptol, *α*- phellandrene, p-cymene, and limonene, which often responded similarly to the bacteria tested with small efficiency (Table 4). The same analysis indicated that *E. coli*, *L. innocua*, and *S. pyogenes* were the most sensitive bacterial strains towards these essential oils' components. Moreover, the assessment of the MIC and MBC of the four essential oils, *E. grandis*, *E. anceps*, *E. camaldulensis*, and *E. obliqua*, was determined. Essential oils from *E. grandis* and *E. anceps* showed higher antibacterial efficacy compared to other essential oils tested (*p* < 0.05) with MIC and MBC ranging from 0.5 to 7 and 2.5 to 20 *μ*L/mL, respectively. While for *E. camaldulensis* and *E. obliqua*, MIC and MBC were found to vary from 3.5 to 50 and 15 to 75 *μ*L/mL, respectively (Table 5).

### 3.4. Antibacterial Activity of HPC–Essential Oil–Based Materials

In this study, essential oils from both *E. grandis* and *E. anceps,* which were the most effective drugs against the bacteria's growth, served for further study of their possible use in making antimicrobial films. To verify their application as tools to make food packaging materials, these two efficacious essential oils were incorporated in HPC to make two films, HPC-Eg and HPC-Ea for *E. grandis* and *E. anceps*, respectively. Films of the white color with thickness ranging from 0.2 to 0.22 mm were obtained. They were then tested for antibacterial activity. In comparison with HPC films, which allowed all bacteria to grow as needed, at all the concentrations used (5–15 *μ*L/mL), the two essential oils in HPC films indicated significant inhibition growth of all bacteria (*p* < 0.05). The LSD test analysis showed that the antibacterial efficiency of the coated films increased with essential oils' concentrations for all bacteria tested. At the concentration of 15 *μ*L/mL, both essential oils have completely inhibited the growth of bacteria, while at low concentrations of 5 and 10 *μ*L/mL, films containing essential oil from *E. grandis* exhibited higher antibacterial activity compared to those with essential oil from *E. anceps* (Table 6) (*p* < 0.05), the only exception was remarked on *K. pneumoniae*, where the two essential oils exhibited similar antibacterial activity (*p* > 0.05). With the LSD test, *S. aureus*, *L. innocua*, and *S. pyogenes* were more sensitive than *E. coli*, *S. typhimurium*, and *K. pneumoniae* towards essential oil–coated films. Films containing Tween 80 did not indicate any significant inhibition on the growth of all bacteria tested.

## 4. Discussion

**Figure 1:**
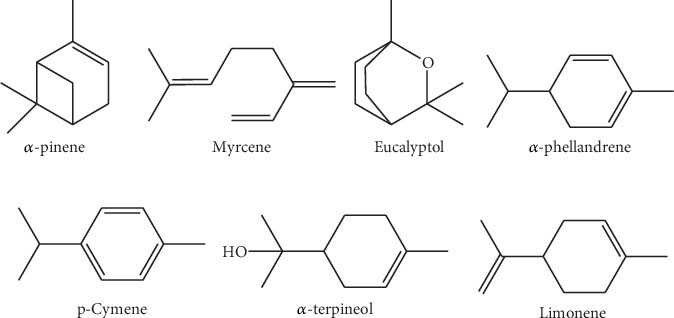
Main chemical components found in oils from various species of eucalyptus.

**Figure 2:**
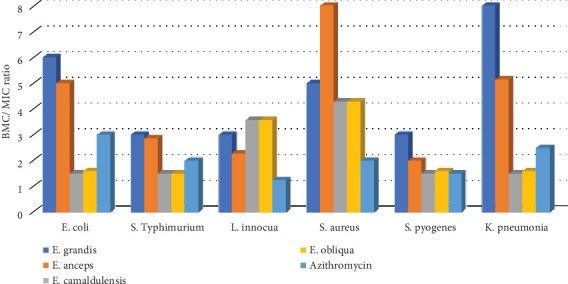
Different MBC/MIC ratios from eucalyptus oils.

The world is confronting different issues hindering humans from progressing as needed. The resistance of bacteria to different drugs, including antibiotics, disinfectants, and films, is among these, and it has been reported several times [11–13]. This problem may constitute a barrier for human beings by affecting their social and economic activities, including industrial food packaging, where the sterility of all used materials is paramount in this field. Indeed, dormant bacteria from nonsterilized materials can revive and contaminate packed goods, causing a danger to consumers. One of the new strategies to avoid this contamination is to use incorporated bioactive drugs in film packaging materials. However, various products for this purpose indicated different side effects [14, 51], and their replacement needs are of interest. The biological activity of essential oils extracted from plant materials has been reported several times [17, 19, 34, 42, 45, 46, 52, 53] and, therefore, can constitute candidates for this purpose. These oils are natural products composed of an unnumbered types of chemicals with concentrations varying from higher to trace amounts. As with other natural products, their toxicities are limited, consequently used as medicines to treat some diseases [16–18]. In this study, essential oils from different eucalyptus species were extracted and evaluated for their antibacterial activity. Their yields (Table 1) were in the range of previous reports [54, 55]. Generally, remarked differences in essential oil yields can be justified by various factors, including season, ecological factors, age of the plants, sampling time, extraction methods, and many others [37–40]. Except for their genetic makeup, eucalyptus materials used in this study had similar exogenous and endogenous influencing factors, and they were similarly treated to get essential oils. Therefore, the only condition that may be the reason for oils' yield variability is the species genotypes. Plant genotype influences carbon allocation and energy use, affecting the biosynthesis of secondary metabolites, including essential oils, and their use speed [52]. Even if these products are used for protection against predators and pollinator attraction, the resilience and stress response of the plant to predators depend on its genotype [56]. Therefore, different eucalyptus species used in this study may respond differently to the production and use of essential oils, explaining the difference in oil yields obtained (Table 1). Moreover, this study assessed the antibacterial activity of these different eucalyptus essential oils to determine species that can be efficient for film packaging materials. Chemical analysis indicated that chemical components varied within eucalyptus species and were in full support of the previous reports highlighting plant species as one of the factors affecting the types and concentration of chemical components in essential oils [38]. The extracted oils contained chemical components classified as terpenoids, alcohol, aldehyde, phenol, ester, and ketone products (Table 2). Previous reports indicated that oils with aldehyde, alcoholic, and phenolic groups are known to exhibit pronounced antimicrobial activities [57–61]. To verify the antimicrobial efficacy of eleven crude essential oils, six bacterial strains; three Gram-positive bacteria, *E. coli*, *L. innocua,* and *S. aureus*; and three Gram-negative bacteria, *S. typhimurium*, *S. pyogenes*, and *K. pneumoniae*, which are known to contaminate foodstuffs and thus to cause different human diseases, including intestinal and respiratory infections [3–7], were used. Four essential oils from *E. grandis*, *E. camaldulensis*, *E. obliqua*, and *E. anceps* exhibited the highest antimicrobial activity against both Gram-positive and Gram-negative bacteria. Among them, oils from *E. grandis* and *E. anceps* were the most effective. In comparison with azithromycin, a known antibiotic, these essential oils exhibited higher antibacterial efficacy against the growth of *S. aureus* for both essential oils, and *S. pyogenes* for essential oil from *E. anceps* (*p* < 0.05). Surprisingly, this study did not show significant antimicrobial activity from *E. crebra*, *E. globulus*, *E. maideni*, *E. melliodora*, *E. microcoris*, *E. polyanthemus*, and *E. viminalis* oils (Table 3), which were mainly composed of a high percentage of eucalyptol (> 57%, Table 2). Hence, individual components' contribution to the remarked antibacterial activity was assessed. Chemicals which was found as the main components of the tested oils, such as *α*-pinene, myrcene, eucalyptol, *α*-phellandrene, p-cymene, *α*-terpineol, and limonene (Figure 1), were used to assess the antibacterial activity against the growth of the selected strains, two Gram-negative (*E. coli* and *S. typhimurium*) and two Gram-positive (*L. innocua* and *S. aureus*) bacteria. These chemical components were found as major components in *E. grandis*, *α*-pinene (39.9%) and p-cymene (25.8%); *E. camaldulensis*, p-cymene (40.1%); *E. obliqua*, *α*-pinene (88.3%); and *E. anceps*, p-cymene (14.1%). Despite its higher concentration in most essential oils tested and exception made on *E. coli*, where it exhibited moderate antibacterial activity, pure eucalyptol did not indicate significant antibacterial activity (Table 4) against the growth of the assessed bacteria, and this corroborated with previous reports [62]. However, *α*-pinene and *α*-terpineol, for example, which were found in small quantities in *E. anceps*, *E. grandis*, and *E. camaldulensis*, compared to the quantity of eucalyptol, indicated significant antibacterial activity on some bacteria (*p* ≥ 0.1). It should be noted here that the observed bioactivities do not necessarily lie in the main components, but minor components should also be highly considered. This study indicated that essential oils from *E. grandis*, *E. anceps*, and *E. camaldulensis*, with a higher number of alcoholic, aldehydic, and ketonic compounds, including *α*-terpineol, cryptone, geranial, piperitone, cis-piperitol, and terpinen-4-ol, even in small quantities, were more efficient against the growth of bacteria than others. According to previous reports [63], these rare components in essential oils may not only strengthen the actions of the major components through positive synergic effects but also can act as an antagonist to toxic major components by exerting their negative synergic effects. In addition, biological activities of *α*-terpineol, terpinen-4-ol, geraniol, (*α*+*β*)-pinene, piperitone, aliphatic alcohols, limonene, and phellandrene have been confirmed by other research findings [62, 64–66]. These reports support our findings and may explain the antibacterial activity obtained from the crude essential oils of *E. grandis*, *E. anceps*, and *E. camaldulensis*. Moreover, in this study, the *α*-pinene was active against the growth of some bacteria despite the lower activity that was previously reported [67–70]. Findings from these authors did not coincide with those reported [62], who found that *α*-pinene exhibited more antibacterial activity than eucalyptol. These research findings [62] corroborated with those obtained in this study and may explain the significant antibacterial activity obtained from *E. grandis* that contained *α*-pinene as its main component (39.9%). The eucalyptol antibacterial activity was also reported [70], and this report contrasted with our findings. All these divergences indicated that the study of antimicrobial activity of essential oils is seriously affected by various factors, including the nature of components in oils, the bacterial strains used during the biological assay, and their behavior towards drugs. Due to their complexity in chemical composition, terpenoids may exhibit their biological activity through synergistic and additive effects of their constituents. Therefore, the interaction between these components may be considered as one of the factors determining the type of biological activity of essential oils. Thus, their mechanism of action may be associated with many components rather than one compound in the oil [71]. Furthermore, the resistance of microbes towards drugs can be taken as another important factor leading to the diversity of biological activity. This study has shown that Gram-positive bacteria, *L. innocua*, *S. aureus*, and *S. pyogenes*, were more sensitive than Gram-negative bacteria, *E. coli*, *S. typhimurium*, and *K. pneumoniae* towards all tested eucalyptus essential oils. This observation is similar to the previous findings [72–75]. The sensibility of Gram-positive bacteria towards essential oils may be associated with the nature of its thick peptidoglycans membrane favoring the least entrance of the drugs through the cell wall and reaching the site of action; as for Gram-negative bacteria, their cells' membranes are more complex with lipopolysaccharides hindering the diffusion of hydrophobic compounds within the cells [75] and thus protect them against these products. While some studies tried to propose the logical way in which essential oils' components destroy the normal functions of microorganisms, the mechanism used by these compounds to hinder the activity of microbes seems not to be well known. However, some tentative explanations have been reported [75–77]. Due to their hydrophobicity and lipophilicity characteristics, essential oils are attracted easily by lipophilic media, thus preferably passing from the aqueous phase into microorganisms' membranes. They fix cell membranes and disrupt them, leading to their fluidity and permeability, allowing them to enter the cells and reach the cytoplasm. In the cytoplasm, these products exert their action by damaging its membrane, coagulating its contents, and creating cell malfunction and death [75–77]. The minimum inhibitory or bactericidal concentrations (MIC/MBC) of the tested oils varied depending on the species of eucalyptus, and this conforms with previous [78–81]. Essential oils from *E. grandis* and *E. anceps* exhibited antibacterial activity with much lower MIC and MBC than oils from *E. camaldulens* and *E. obliqua*. *S. aureus* was the most sensitive to all tested essential oils. It required a MIC of 0.5 *μ*L/mL for *E. grandis* and *E. anceps* essential oils and 3.5 *μ*L/mL for those from *E. camaldulensis* and *E. obliqua*, while *S. typhimurium* was the most resistant bacteria towards the tested oils with MIC of 5, 7, 50, and 50 *μ*L/mL for *E. grandis*, *E. anceps*, *E. camaldulensis*, and *E. obliqua*, respectively. For the most sensitive bacteria strain, *S. aureus*, the lowest MIC for *E. grandis* and *E. anceps* (0.5 *μ*L/mL) was 7 times smaller than the values of the lowest MIC for *E. camaldulensis* and *E. obliqua* (3.5 *μ*L/mL). Therefore, the antibacterial activity from *E. camaldulensis* and *E. obliqua* was much lower than that of *E. grandis* and *E. anceps*. Azithromycin used as a positive control has indicated MIC varying from 2 to 4 *μ*L/mL. For *S. aureus*, the MIC of azithromycin was 4 times greater than that of *E. grandis* and *E. anceps* oils. The ratio between MBC and MIC (MBC/MIC) indicated which essential oil can kill or delay the growth of the bacterial strains. A drug can be considered as containing bactericidal action if the ratio MBC:MIC is equal to or smaller than 4 and bacteriostatic effect if this ratio is greater than 4 [82, 83]. Azithromycin and essential oils from *E. camaldulensis* and *E. obliqua* indicated bactericidal effect against all tested bacteria, while essential oils from *E. grandis* and *E. anceps* did it on *S. typhimurium*, *L. innocua*, and *S. pyogenes* (Figure 2). These observations may be explained by diverse and different components in the oils that may act differently and by the type of strains used. Besides, the higher antibacterial activity from *E. grandis* and *E. anceps* may be used to test their possible application in making bioactive film materials. In this study, these oils were incorporated into the films to assess their ability to persist with antimicrobial activity in solid media. Results indicated that these essential oils increased the ability of the films to resist the development of strains contaminating foods. These two essential oils indicated their efficacy in inhibiting the growth of *E. coli*, *S. typhimurium*, *L. innocua*, *S. aureus*, *S. pyogenes,* and *K. pneumoniae*, which are cultured in cellulosic films. The higher antibacterial activity of *E. anceps* may be due to the high number of alcoholic components it contains, including cis-piperitol, terpinene-4-ol, cis-p-2-menthol-1-ol, cis-terpineol, trans-piperitol, and geraniol. It was also reported that alcoholic and phenolic terpenes have more antimicrobial activity than the other constituents [84], and this is corroborated by the present findings. In fact, in this study, essential oils from both eucalyptus species indicated the presence of alcoholic components, which may be responsible for the remarked higher antibacterial activity. To be effective, essential oils' active compounds must be released from the films and reach the bacteria's membranes. Because of the possible interaction between antimicrobial agents and the polymer matrix composing the film, the release of these products depends not only on their chemical nature but also on the nature of the film [77]. Knowing that the oils' active ingredients contain hydroxyl groups, mainly alcoholic and phenolic substances, films composed of HPC can easily release active ingredients, allowing them to reach the lipophilic bacteria membranes. Moreover, due to its physicochemical properties allowing the interaction with water, temperature, and moisture, HPC polymer matrix can swell and allow easy diffusion of essential oils through the matrix [85]. The released active ingredients reach bacteria and change their capability to grow and multiply in the films. This assumption may be used to further study the possibility of making films coated with essential oils that can be a new alternative for antimicrobial packaging materials. The development of antimicrobial active film packaging materials with essential oils may be of interest due to their currently known edibility and harmlessness characteristics for living organisms. However, the toxicological profile of these products should be carefully studied before any further application.

## 5. Conclusion

Besides their known uses in medicine, this study showed that essential oils can also be exploited for other purposes, including the manufacture of safe films, which are needed for making safe packaging materials. As some of the aromatic plants are edible, the use of plant extracts for food preservation can be an advantage for food safety and consumers' interests. Results from essential oils of *E. grandis* and *E. anceps* were promising and indicated that plant materials can make a difference in discovering new active ingredients. However, considering the complexity of essential oils, clarification on the real active constituents, their action modes, and their toxicological profiles, the study of these products is still far from being exhausted before their safe application.

## Figures and Tables

**Table 1:** Yields of eucalyptus essential oils of Ruhande Arboretum.

**Species**	**AN**	**CA**	**GR**	**GL**	**ME**	**MI**	**OB**	**CR**	**PO**	**VI**	**MA**
Yields (%)	0.47	0.62	0.72	2.2	1.26	2.12	0.25	1.91	1.02	0.91	1.25

*Note*: AN: *E. anceps*, CA: *E. camaldulensis*, GR: *E. grandis*, GL: *E. globulus*, ME: *E. melliodora*, MI: *E. microcorys*, OB: *E. obliqua*, CR: *E. crebra*, PO: *E. polyanthemus*, VI: *E. viminalis*, MA: *E. maideni*.

**Table 2:** Chemical composition of 11 essential oils of eucalyptus species.

**Components**	**RF**	**KI**	**Identification methods**	**Relative %**										
				**AN**	**CA**	**GR**	**GL**	**ME**	**MI**	**OB**	**CR**	**PO**	**VI**	**MA**
Diisopropyl ketone	2.83	782	KI, MS			1.5		2.0	1.3		0.2	0.6	0.3	0.9
3-Thujene	3.52	928	KI, MS	2.7	4.5		0.4							
*α*-Pinene	3.58	937	KI, AS, MS		3.0	39.9	15.8	9.6	29.6	89.3	3.9	4.2	3.2	6.8
Sabinene	4.58	975	KI, MS			0.1								
*β*-Pinene	4.63	979	KI, AS, MS	0.1	0.2	0.8	0.6		0.1	0.4	1.6	0.1	0.1	0.20
*β*-Myrcene	5.78	990	KI, AS, MS	11.1	1.6		0.5	0.2			0.2	2.1	0.1	1.30
*α*-Phellandrene	6.06	1004	KI, AS, MS	1.7	0.6							0.2		0.1
*α*-Terpinene	5.97	1017	KI, MS		0.4									
p-Cymene	6.41	1026	KI, AS, MS	14.1	40.1	25.8	0.1	0.2	2.0	0.5	0.1	2.1	0.4	0.4
Limonene	6.50	1029	KI, AS, MS	0.3	2.3	2.7	3.6	2.6	2.4	1.1	2.4	2.4	3.0	4.6
*β*-Phellandrene	6.71	1030	KI, AS, MS	4.7	1.9					0.2				
Eucalyptol	6.80	1033	KI, AS, MS	4.9	18.2	14.2	70.4	78.0	57.1	5.5	84.6	80.7	88.3	74.8
*γ*-Terpinene	8.52	1061	KI, MS	0.2	1.0	7.9	0.6							
Terpinolene	8.73	1090	KI, AS, MS	2.8	4.6				0.7			0.5	0.1	0.2
*β*-Linalool	11.69	1098	KI, MS	1.7										0.1
cis)-p-(2-menthen)-1-ol	16.81	1110	KI, MS	8.3	0.6				0.6				0.1	
Terpinen-4-ol	17.47	1179	KI, MS	5.7	5.6									
*α*-Terpineol	18.70	1190	KI, MS	1.6	2.2	2.7								7.1
cis-Piperitol	18.76	1183	KI, MS	4.5	0.1									
trans-Piperitol	18.99	1196	KI, MS	2.0	0.3									
Cryptone	23.82	1229	KI, MS		6.1									
Piperitone	23.89	1252	KI, MS	10.5										
Geraniol	23.99	1255	KI, AS, MS	19.9										
Geranial	24.38	1266	KI, AS, MS	0.1	0.7	1.1								
Bornyl acetate	24.42	1287	KI, MS				1.7	3.4	2.7		3.1	3.8	2.0	
*α*-Terpenyl acetate	24.54	1349	KI, AS, MS		3.2								0.1	
Spathulenol	26.50	1576	KI, MS					0.8						
*α*-Terpineol acetate	26.65	1580	KI, MS				3.0	1.0			0.2			
Total peak area (%)				96.9	97.2	96.7	96.7	96.8	96.5	97.0	97.3	96.7	97.7	96.2

*Note*: AN: *E. anceps*, CA: *E. camaldulensis*, GR: *E. grandis*, GL: *E. globulus*, ME: *E. melliodora*, MI: *E. microcorys*, OB: *E. obliqua*, CR: *E. crebra*, PO: *E. polyanthemus*, VI: *E. viminalis*, MA: *E. maiden*.

*Abbreviations*: AS: analytical standard, KI: Kovats Index, MS: mass spectrometry, RT: retention time.

**Table 3:** Inhibition diameters (mm) of essential oils from 11 different species of eucalyptus against pathogens.

**Drugs tested**	**Microorganisms and inhibition diameter (** **m** **e** **a** **n** ± **SDin mm)**					
	***E. coli***	***S. typhimurium***	***L. innocua***	***S. aureus***	***S. pyogenes***	***K. pneumoniae***
*E. viminalis*	8.2 ± 1.2	7.7 ± 1.1	8.8 ± 1.1	8.2 ± 1.2	8.2 ± 2.1	10.2 ± 2.1
*E. maiden*	11.4 ± 1.4	11.1 ± 1.5	12.2 ± 2.4	10.2 ± 1.5	12.1 ± 3.0	13.6 ± 1.1
*E. melliodora*	9.8 ± 2.5	8.2 ± 1.0	10.5 ± 1.5	11.2 ± 2.0	9.3 ± 1.2	15.4 ± 2.2
*E. grandis*	22.6 ± 2.7^∗^	17.8 ± 1.2	23.1 ± 2.0	35.3 ± 2.2^∗∗^	23.4 ± 2.4^∗^	20.5 ± 2.1^∗^
*E. camaldulensis*	13.2 ± 1.1	11.3 ± 1.1	18.4 ± 1.6	24.4 ± 1.3^∗^	12.4 ± 2.5	15.2 ± 1.3
*E. obliqua*	13.3 ± 2.3	12.2 ± 2.4	16.6 ± 1.1	20.3 ± 2.2	12.5 ± 1.1	16.2 ± 3.0
*E. globulus*	9.3 ± 1.5	9.5 ± 2.3	11.3 ± 2.3	12.2 ± 1.2	11.7 ± 2.0	13.4 ± 1.2
*E. microcoris*	8.8 ± 1.1	8.4 ± 1.5	9.3 ± 1.5	8.4 ± 1.5	8.3 ± 2.1	10.9 ± 3.0
*E. polyanthemus*	12.5 ± 1.1	10.1 ± 1.0	11.2 ± 2.1	17.3 ± 2.2	9.9 ± 2.3	10.7 ± 1.2
*E. anceps*	19.3 ± 1.8	15.5 ± 1.1	21.2 ± 1.4	30.3 ± 3.0^∗∗^	33.3 ± 2.0^∗∗^	17.4 ± 1.1^∗^
*E. crebra*	10.2 ± 1 0.3	9.7 ± 2.1	11.4 ± 1.1	13.2 ± 2.3	9.2 ± 1.1	11.3 ± 1.4
Azithromycin	23.7 ± 1.2	30.3 ± 1.8	28.7 ± 3.0	24.2 ± 1.2	25.5 ± 3.1	19.3 ± 3.1

*Note*: *p* compared to azithromycin: (i) ⁣^∗∗^ < 0.05, (ii) ⁣^∗^ > 0.05.

*Abbreviation*: SD: standard deviation.

**Table 4:** Antibacterial activity of some pure compounds found in eucalyptus essential oils against some bacteria.

**Drugs tested**	**Inhibition diameter (** **m** **e** **a** **n** ± **SDin mm)**					
	***E. coli***	***S. typhimurium***	***L. innocua***	***S. aureus***	***S. pyogenes***	***K. pneumoniae***
*α*-Pinene	25.4 ± 2.2^∗^	12.4 ± 2.2	17.8 ± 3.3	13.8 ± 3.1	19.7 ± 2.1	14.7 ± 1.1
Myrcene	6.2 ± 1.4	6.9 ± 1.4	6.7 ± 1.8	6.6 ± 1.5	6.9 ± 1.1	6.7 ± 0.9
Eucalyptol	12.6 ± 1.7	7.8 ± 1.4	8.6 ± 1.3	7.8 ± 1.4	9.2 ± 1.2	7.7 ± 1.6
*α*-Phellandrene	6.9 ± 1.8	7.8 ± 1.4	10.4 ± 2.1	9.4 ± 1.4	11.7 ± 1.5	8.7 ± 2.0
p-Cymene	9.8 ± 1.3	8.5 ± 1.5	9.9 ± 1.3	6.5 ± 1.6	11.2 ± 1.7	9.5 ± 1.9
*α*-Terpineol	24.5 ± 3.0^∗^	17.3 ± 2.3	24.8 ± 2.4^∗^	21.4 ± 2.4^∗^	24.9 ± 1.6^∗^	20.2 ± 1.5^∗^
Limonene	15.6 ± 2.2	6.6 ± 1.4	9.5 ± 1.4	6.5 ± 1.1	11.7 ± 1.8	9.5 ± 1.7
Azithromycin	23.7 ± 1.2	30.3 ± 1.8	28.7 ± 1.2	24.2 ± 1.2	25.5 ± 3.1	19.3 ± 3.1

*Note*: *p*, in comparison with azithromycin: ⁣^∗^*p* > 0.05.

*Abbreviation*: SD: standard deviation.

**Table 5:** Minimal inhibitory concentration (MIC) and minimal bactericidal concentration (MBC) of different essential oils from *E. grandis*, *E. anceps*, *E. camaldulensis*, and *E. obliqua* leaves.

**Microorganisms**		**Essential oils and required MIC and MBC (*μ*L/mL)**				
		***E. grandis***	***E. anceps***	***E. camaldulensis***	***E. obliqua***	**Azithromycin**
*E. coli*	MIC	0.625	0.75	20.0	25.0	2.00
	MBC	3.75	3.75	30.0	40.0	6.00
*S.* Typhimirium	MIC	5.00	7.0	50.0	50.0	3.00
	MBC	15.0	20.0	75.0	75.0	6.00
*L. innocua*	MIC	1.25	1.75	7.00	7.0	4.00
	MBC	3.75	4.00	25.0	25.0	5.00
*S. aureus*	MIC	0.50	0.50	3.50	3.50	2.00
	MBC	2.50	4.00	15.0	15.0	4.00
*S. pyogenes*	MIC	1.25	1.75	20.0	25.0	4.00
	MBC	3.75	3.50	30.0	40.0	6.00
*K. pneumoniae*	MIC	0.625	0.875	20.0	25.0	2.00
	MBC	5.00	4.50	30.0	40.0	5.00

**Table 6:** Antibacterial activity of HPC-based films containing *E. grandis* and *E. anceps* essential oils.

**Films**	**Quantity of drugs (*μ*L/mL)**	**Percentage of inhibition (** **m** **e** **a** **n** ± **SD )**					
		***E. coli***	***S. typhimurium***	***L. innocua***	***S. aureus***	***S. pyogenes***	***K. pneumoniae***
HPC	—	0.0 ± 0.0	0.0 ± 0.0	0.0 ± 0.0	0.0 ± 0.0	0.0 ± 0.0	0.0 ± 0.0

HPC-Eg	5	67.6 ± 1.1^∗∗∗^	71.3 ± 0.7^∗∗∗^	83.7 ± 1.3^∗∗∗^	85.6 ± 0.9^∗∗∗^	79.1 ± 1.7^∗∗∗^	68.6 ± 2.1^∗∗∗^
	10	77.3 ± 1.3^∗∗^	81.7 ± 1.2^∗∗^	95.4 ± 1.4^∗∗^	95.7 ± 1.1^∗∗^	90.4 ± 2.0^∗∗^	75.2 ± 1.3^∗^^+^
	15	100.0 ± 0.0	100.0 ± 0.0	100.0 ± 0.0	100.0 ± 0.0	100.0 ± 0.0	100.0 ± 0.0

HPC-Ea	5	55.7 ± 2.2^∗∗∗^	65.5 ± 1.5^∗∗∗^	71.2 ± 1.2^∗∗∗^	73.2 ± 0.7^∗∗∗^	69.9 ± 1.9^∗∗∗^	60.2 ± 1.9^∗∗∗^
	10	60.3 ± 1.7	69.7 ± 1.0	80.3 ± 2.3	80.9 ± 1.9	81.3 ± 2.1	76.9 ± 2.3^∗^^+^
	15	100.0 ± 0.0	100.0 ± 0.0	100.0 ± 0.0	100.0 ± 0.0	100.0 ± 0.0	100.0 ± 0.0

HPC-T80	5%	2.3 ± 0.4^∗^	3.0 ± 1.0^∗^	5.9 ± 0.9^∗^	2.9 ± 0.8^∗^	3.6 ± 1.1^∗^	1.5 ± 0.6^∗^

*Note*: HPC: hydroxy-propyl cellulose film, HPC-Eg: hydroxy-propyl cellulose film containing *E. grandis* essential oil, HPC-Ea: hydroxy-propyl cellulose film containing *E. anceps* essential oil, HPC-T80: hydroxy-propyl cellulose film containing Tween 80.

⁣^∗∗∗^Significant antimicrobial activity in comparison with HPC film, *p* < 0.005; ⁣^∗∗^HPC-Eg is more active than HPC-Ea, *p* < 0.05; ⁣^∗^no significant difference between activity from HPC-T80 and HPC itself, *p* > 0.05; and ⁣^∗^^**+**^no significant difference between HPC-Ea and HPC-Eg, *p* > 0.05.

## Data Availability

The voucher specimens of the plant materials used can be seen by a physical visit to the Herbarium of the CoEB, Rwanda.
